# HSV-1 virions and related particles: biogenesis and implications in the infection

**DOI:** 10.1128/jvi.01076-24

**Published:** 2025-02-03

**Authors:** Maria Kalamvoki

**Affiliations:** 1 Department of Microbiology, Molecular Genetics, and Immunology, University of Kansas Medical Center 21638 , Kansas City, Kansas, USA; Universiteit Gent, Merelbeke, Belgium

**Keywords:** extracellular vesicles, L-particles, tetraspanins, ESCRT, ICP0, ICP34.5, autophagy, innate immunity, STING, virus egress

## Abstract

Virion formation and egress are sophisticated processes that rely on the spatial and temporal organization of host cell membranes and the manipulation of host machineries involved in protein sorting, membrane bending, fusion, and fission. These processes result in the formation of infectious virions, defective particles, and various vesicle-like structures. In herpes simplex virus 1 (HSV-1) infections, virions and capsid-less particles, known as light (L)-particles, are formed. HSV-1 infection also stimulates the release of particles that resemble extracellular vesicles (EVs). In productively infected cells, most EVs are generated through the CD63 tetraspanin biogenesis pathway and lack viral components. A smaller subset of EVs, generated through the endosomal sorting complexes required for transport (ESCRT) pathway, contains both viral and host factors. Viral mechanisms tightly regulate EV biogenesis, including the inhibition of autophagy—a process critical for increased production of CD63+ EVs during HSV-1 infection. Mutant viruses that fail to suppress autophagy instead promote microvesicle production from the plasma membrane. Additionally, the viral protein ICP0 (Infected Cell Protein 0) enhances EV biogenesis during HSV-1 infection. The different types of particles can be separated by density gradients due to their distinct biophysical properties. L-particles and ESCRT+ EVs display a pro-viral role, supporting viral replication, whereas CD63+ EVs exhibit antiviral effects. Overall, these studies highlight that HSV-1 infection yields numerous and diverse particles, with their type and composition shaped by the ability of the virus to evade host responses. These particles likely shape the infectious microenvironment and determine disease outcomes.

## HSV-1 INFECTION AND CLINICAL MANIFESTATIONS

Herpes simplex virus 1 is a neurotropic human pathogen that has infected over 67% of the global population (1). It initially infects mucosal epithelial cells, then enters sensory neurons at the primary infection site, and establishes latent reservoirs in the peripheral ganglia containing infected sensory neurons (2, 3). Clinically, HSV-1 often presents with blisters, sores, or ulcers in the orofacial and genital regions. The virus can also damage the cornea and lead to stromal keratitis, making it the leading infectious cause of blindness in developed countries (4–8). In rare cases, HSV-1 can replicate in the brain resulting in herpes simplex encephalitis (HSE)—a severe inflammatory brain condition with a mortality rate of 70% if left untreated (9). Evidence also implicates HSV-1 in neurodegenerative diseases, including Alzheimer’s disease (AD) (10–20). This may result from subclinical (or “silent”) reactivation and replication of the virus in the brain, leading to neuronal loss and inflammation, contributing to neurodegeneration. Supporting this idea, two independent population studies found a correlation between elevated anti-HSV-1 IgM levels (indicating primary HSV-1 infection) in elderly, dementia-free, individuals and an increased risk of developing AD (21). Additionally, a positive correlation has been observed between the anti-HSV-1 IgG avidity index (a marker of frequent HSV-1 reactivation) and neurodegeneration (22). Some studies have also attempted to address whether HSV-1 reactivation occurs directly within the central nervous system (CNS) or if the virus translocates to the CNS following reactivation and replication in the trigeminal ganglia (23). Latent HSV-1 has been detected in postmortem brain samples from both AD patients and healthy elderly controls, suggesting that an HSV-1 reservoir likely exists in the human brain (24). These findings underscore the urgent need to determine whether HSV-1 is a causal factor or an “opportunistic passenger” of neurodegeneration.

Following the entry of HSV-1 into host cells, viral gene transcription, genome replication, and the assembly of the newly made capsids occur in the nucleus of the infected cells. The nucleocapsids bud into the inner nuclear membrane, acquiring a primary envelope as the virions pinch off into the perinuclear space. The capsids are then released into the cytoplasm following fusion of the primary envelope with the outer nuclear membrane. Tegument proteins associate with the nucleocapsids in both the nucleus and the cytoplasm. Cytoplasmic envelopment occurs in structures resembling the trans-Golgi network (TGN) that contain TGN-specific proteins and lipids (1, 25–31). HSV-1 capsids do not colocalize with lysosomes, the ER, or other Golgi compartments. An alternative model proposes that envelopment occurs in endocytic tubules derived from the plasma membrane, a process dependent on Rab5 and Rab11 (32). These findings suggest that HSV-1 virion envelopment is a versatile process, and perhaps, the site of secondary envelopment is determined by both growth conditions as well as the duration of time that mature viral enveloped proteins are retained in the different membranous compartments.

During HSV-1 infection, in addition to infectious HSV-1 virions, a variety of non-infectious particles are produced. Some of these particles resemble virions in composition, as they contain both viral tegument and envelope proteins, but they lack nucleocapsid, which distinguishes them from their infectious counterparts. These particles are known as light (L) particles, as termed due to density gradient dynamics, and will float in lighter density fractions compared with the heavy (H) particles representing intact virions (33–38). Other particles released from HSV-1-infected cells are composed exclusively of host factors. These particles resemble extracellular vesicles (EVs), and because they are highly enriched in the tetraspanin CD63, they are also known as CD63+ EVs (39). HSV-1-infected cells also release chimeric particles composed of both viral and host components. These particles contain elements of the endosomal sorting complexes required for transport (ESCRT) and are thus termed ESCRT+ EVs (39). The composition of these particles depends on the ability of HSV-1 to evade host antiviral responses (40). In the CNS, HSV-1 can infect both neurons and glial cells. It can replicate most efficiently in neurons, less efficiently in astrocytes, and only minimally in microglia, which mount robust inflammatory responses following HSV-1 infection (41, 42). It is thus important to characterize the populations of particles released after infecting the different neuronal cell types, and their impact on the latent reservoir of the virus. Furthermore, it is critically important to determine whether these particles trigger chronic antiviral responses in recipient CNS cells and contribute to neurodegeneration.

## DOMINANT POPULATIONS OF PARTICLES RELEASED BY HSV-1-INFECTED CELLS

Although the virus replication cycle is traditionally viewed as a well-defined sequence of events, a growing body of evidence highlights extreme heterogeneity at different stages of this process. Variations in genome packaging, virion morphogenesis, and egress can result from environmental factors, cell type differences, and antiviral defenses. These factors collectively lead to the formation of pleomorphic, semi-infectious, or non-infectious particles (43). Because some particles have reduced or no infectivity, different viruses exert varying particle-to-PFU ratios. Some viruses, like bacteriophages, have a ratio close to 1, indicating that nearly every particle is infectious. Under optimal propagation conditions, this ratio is 10 for HSV, 20–50 for influenza, 40,000 for VZV, 10^4^–10^6^ for SARS-CoV-2, and as high as 10^7^ for some HIV-1 variants (44–48). Additionally, a compensatory mechanism for virion heterogeneity has been proposed via the formation of cloaked virions (49–54). This mechanism involves multiple virions packaged together as a single unit (or “cloaked”) inside microvesicles, promoting the spread of multiple heterogeneous virions collectively (49, 51–54). This mode of spread could compensate for defective or incomplete genomes and perhaps promote virus evolution. When viral particles infect in clusters, they are also likely to increase the multiplicity of infection. Picornaviruses, for example, appear to package multiple viral particles into microvesicles, whereas VSV, polioviruses, poxviruses, and influenza aggregate to form multi-virion infectious units (43, 49–55).

Non-infectious, virus-like particles were initially considered byproducts of the cell culture systems and irrelevant to the natural infection. However, recent data indicate that such particles also arise *in vivo*. For example, nasopharyngeal samples from influenza A patients harbored RNA species containing large internal deletions but retained packaging signals similar to defective interfering particles (DIPs) produced *in vitro* (56, 57). Similar findings have been reported for other respiratory viruses, including RSV, MERS, and SARS-CoV-2 (58, 59). Further, serum from Dengue virus-infected patients contained RNA fragments with intact regulatory and packaging elements at the 3′ and 5′ ends, comparable with DIPs (60).

**Fig 1:**
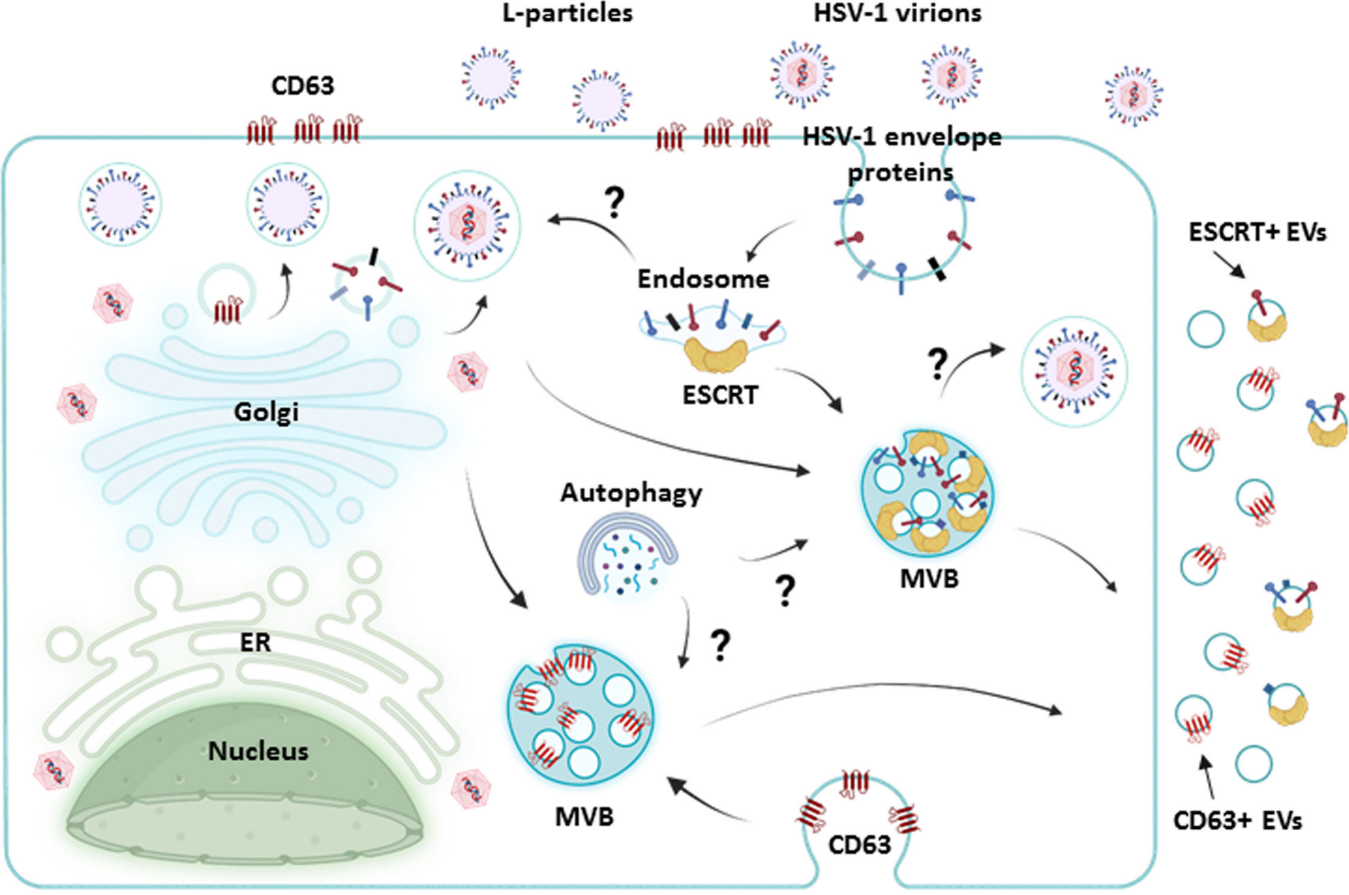
Membrane trafficking pathways involved in HSV-1 egress and the biogenesis of related particles. HSV-1 capsids assemble into the nucleus, then acquire the primary envelop from the inner nuclear membrane, de-envelop in the outer nuclear membrane, and released into the cytoplasm for secondary envelopment. The TGN network appears to be the major source of membranes for cytoplasmic HSV-1 envelopment. In addition, endocytic tubules, regulated by the Rab5 (early endosomes) and Rab11 (recycling endosomes) GTPases, participate in HSV-1 envelopment. Also, multivesicular bodies may provide ESCRT components for HSV-1 envelopment, but their exact roles remain unclear. Similar pathways are likely used for the production of L-particles, although L-particle release is independent of mature virion release. Viral late gene expression and virion translocation to the cytoplasm stimulate biogenesis of CD63+ EVs. CD63 can be sorted into endosomes through the plasma membrane and the TGN. A less abundant population of EVs carries ESCRT components along with selected viral factors. Sorting of viral factors and ESCRT components to endosomes likely occurs through the TGN and/or the plasma membrane. HSV-1 effectively blocks autophagy and components of this pathway are likely diverted for exocytosis.

In the case of HSV-1, L-particles are virus-like particles that lack the capsid and viral DNA, rendering them non-infectious (33–38). Infected cells produce similar amounts of both infectious virions and L-particles (33–38). The exact mechanisms of assembly and exit of these L-particles remain to be elucidated. For virions, the envelope is acquired through the TGN, and enveloped virions are transported to the plasma membrane, exiting the cell by fusion of the membranous vehicle with the plasma membrane (1, 25–31). Some studies have also implicated multivesicular bodies (MVBs) and other membranes from the endocytic pathway in the final envelopment (32, 61). This suggests that the process of secondary envelopment in HSV-1 is complex, with the TGN-mediated egress pathway being the dominant course and resulting in the production of a diverse array of particles (Fig. 1). Most studies show that L-particles and virions form both simultaneously within the same envelopment compartments. However, studies using a temperature-sensitive mutant that produces capsids unable to package viral DNA or escape the nucleus demonstrated that L-particles can still be produced and released without mature virions (38, 62). This suggests that the formation of enveloped tegument structures (L-particles) can occur independently of full virion assembly and maturation. L-particles are present in infections caused by all alphaherpesviruses, and there is evidence for production *in vivo* as well. For example, significant amounts of L-particles were detected in the nasal mucosa of swine infected with pseudorabies virus (63).

**Fig 2:**
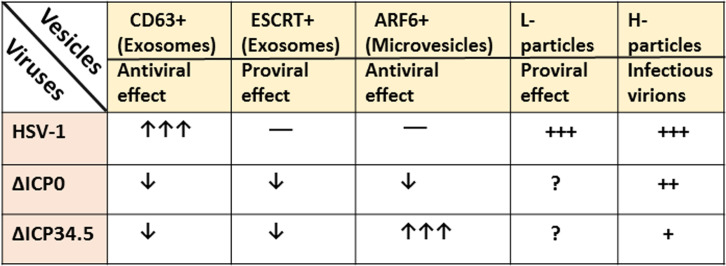
Major populations of particles identified in HSV-1 productively infected cells. HSV-1 infectious virions (H-particles) are produced in a similar number as the non-infectious particles (L-particles) (38). In addition, HSV-1 infection promotes biogenesis of CD63+ EVs compared with uninfected cells (39, 64, 65). HSV-1 infection also utilizes the ESCRT machinery for primary and secondary envelopment, but the abundance of ESCRT+ EVs is comparable between infected and uninfected cells. Finally, HSV-1 infection does not promote microvesicle production. Unlike the WT virus, a ΔICP34.5 displayed increased production of microvesicles through the plasma membrane, but biogenesis of CD63+ and ESCRT+ EVs was substantially reduced (40). ΔICP34.5 virus produces fewer infectious particles compared with the WT virus, whereas quantification of the L-particles is pending. Finally, a ΔICP0 virus infection produces fewer infectious particles compared with the WT virus infection, and all other particle populations are significantly reduced (40).

Recently, we described that in addition to the L-particles, HSV-1-infected cells also produce particles that resemble extracellular vesicles (EVs) (39, 64–66). A summary of particle populations identified in HSV-1-infected cells and after infection with mutant viruses is depicted in Fig. 2. To date, all studies have been conducted in human immortalized fibroblasts or spontaneously immortalized human retinal epithelial cells, where the virus productively replicates. Cancer cells may not be ideal for studying HSV-1 infection and released particles because they alter EV biogenesis pathways to ensure that the tumor microenvironment receives a constant flow of nutrients (67, 68). Similarly, non-human cells may not accurately reflect HSV-1 infection dynamics, as they are not the natural host, and the virus may not effectively counteract antiviral responses. We discovered that productively infected cells release 10–100 times more EVs than the total plaque-forming units (PFU) of the virus from the same culture (39, 64, 65). The predominant EV population was enriched in the tetraspanin CD63, with only trace amounts of other tetraspanins such as CD81 and CD9 (39). Importantly, the CD63+ EVs lacked viral components (39). Infected cells produced about twice as many CD63+ EVs as uninfected cells within 48 h (39). CD63, a member of the tetraspanin family of proteins, forms microdomains rich in cholesterol, gangliosides, and associated proteins. This tetraspanin web regulates the trafficking, post-translational modifications, and functions of these associated proteins (69–71). Major partners of CD63 include integrins, other tetraspanins, adaptor proteins, enzymes (such as metalloproteinases and kinases), and signaling receptors (69–71). CD63 is primarily localized within late endosomes, including multivesicular endosomes and lysosomes, making it abundant in exosomes (71–74). Like other type I integral lysosomal membrane proteins, CD63 bears a YXXØ sorting motif in its cytoplasmic tail, essential for plasma membrane endocytosis and targeting from the TGN to lysosomes (69–71). Although the plasma membrane-to-endosome pathway of CD63 is dominant, evidence suggests a direct TGN-to-endosome pathway exists. These pathways could link the biogenesis of CD63+ EVs with virion production. In fact, the stimulation of CD63 exocytosis during HSV infection requires late gene expression, capsid assembly in the nucleus, and possibly primary envelopment (39, 65). Consistent with this, we observed that the replication-deficient virus ΔICP8, and the capsid assembly-deficient mutant ΔUL18 did not promote the production of CD63+ EVs (39, 65). However, secondary envelopment in the cytoplasm is not required for CD63 exocytosis, as envelopment-deficient mutants ΔUL36 and ΔUL37 stimulated CD63 exocytosis to the same levels as the WT virus (39, 65). These findings suggest that partial acquisition of the tegument and primary envelopment are sufficient to stimulate CD63 exocytosis.

Additionally, we identified a less dominant EV population carrying components of the ESCRT machinery and selected viral factors, mainly envelope and tegument proteins, which we termed ESCRT+ EVs (39). Historically, the ESCRT system has been described as a network of complexes that cooperatively sort integral membrane proteins modified by ubiquitin. It consists of more than 30 gene products that form molecular machines for various cellular activities, including cytokinesis, nuclear envelope reformation, membrane repair, and autophagy (75–80). Due to these broad functions, viruses often hijack ESCRT for budding (81). Additionally, there is evidence that ESCRT facilitates HSV-1 primary and secondary envelopment, with multiple viral proteins interacting with ESCRT components (25, 27, 61, 82–89). Despite similar numbers of ESCRT+ EVs between infected and uninfected cells, qualitative differences were observed, including that the ESCRT+ EVs from infected cells lacked certain ESCRT components. Also, despite some similarities between the ESCRT+ EVs and L-particles, we identified some substantial differences. Although the L-particles are highly enriched in tegument proteins UL46, VP22, and the gM envelope protein, these proteins are absent from ESCRT+ EVs (39, 65, 90). Additionally, ESCRT+ EVs differ in density compared with the L-particles (detailed below). Therefore, it is a possibility that ESCRT+ EVs and L-particles are not directly related. Alternatively, they might share the same biogenesis pathway, with ESCRT+ EVs containing fewer viral components, potentially representing lighter L-particles. Another possibility is that viral components may not actually be present in the ESCRT+ EVs but constitute a separate population of particles with the same density. Notably, CD63+ EVs and ESCRT+ EVs are distinct, as CD63 is absent from ESCRT+ EVs and vice versa.

Given that cells can produce different populations of EVs, other types of particles are likely to be released by infected cells. It is important to note that factors co-fractionating in density gradients are not necessarily components of the same EV population. Further research is underway to better define the populations of particles released by infected cells. The abovementioned data pertain to productively infected cells. In cells where HSV-1 is unable to evade antiviral responses, such as autophagy, different populations of EVs are likely produced, as discussed below. Overall, our studies demonstrate that a viral infection results in the release of both infectious and a plethora of non-infectious particles, some of which exhibit features derived from both the virus and the host.

## ROLE OF HSV-1 GENES IN THE BIOGENESIS OF DIFFERENT PARTICLE POPULATIONS

Although the coordinated actions of viral products are essential for the formation of virions and L-particles, the role of viral products in EV biogenesis is an emerging area of study. Given the crosstalk between autophagy and exocytosis, we examined how viral genes involved in autophagy inhibition affect EV biogenesis. The infected-cell protein 34.5 (ICP34.5), encoded by a gamma-1 or leaky late gene of HSV-1, is detectable as soon as 3 h post-infection. It is primarily characterized as a neurovirulence factor essential for HSV-1 infection of neurons *in vivo* (91–99). Consistently, studies have shown that an ICP34.5-null virus is neuroattenuated in mouse brains (91–99). ICP34.5 has complex functions that vary depending on its localization, which depends on the stage of the infection and the cell type (91–99). Initially, ICP34.5 was found to block dsRNA-dependent protein kinase R (PKR) signaling (91, 92). PKR activation can cause translational arrest by phosphorylating the translation initiation factor 2 subunit α (eIF2α). ICP34.5 counters this by recruiting protein phosphatase 1α (PP1α) to dephosphorylate eIF2α, preventing translational shutoff (91, 92). Beyond PKR inhibition, ICP34.5 has been found to directly inhibit autophagy by binding Beclin-1, a key player in autophagosome formation (from autophagosome nucleation to their maturation) (97). Considering that endolysosomal CD63 bridges autophagic turnover and exosomal secretion, we investigated how CD63+ EVs production is affected by an ICP34.5-null virus (100). As previously discussed, cells productively infected with the WT virus produce twice as many EVs as uninfected cells, most of which are CD63+. These EVs are also enriched in LAMP-1 (lysosomal-associated membrane protein 1) and lipidated LC3, both essential components of the autophagy pathway. Exocytosis of these factors during WT virus infection may represent a complementary mechanism by which the virus evades autophagy. Conversely, cells infected with the ΔICP34.5 virus produce a large number of EVs that lack CD63 and other tetraspanins like CD81 and CD9 (40). Additional characterization revealed that these EVs do not undergo exocytosis through the lysosomal pathway, as LAMP-1 and lipidated LC3 are degraded through the autophagolysosomal pathway (40). Instead, the EVs are enriched in plasma membrane-associated factors, including the small GTPases Rho A and ARF6, as well as Annexin A1 (40). These factors serve as markers of plasma membrane-derived extracellular vesicles, also known as microvesicles. It appears that when HSV-1 cannot evade autophagy, CD63 diverts cargo for degradation instead of exocytosis, leading to massive EV production via the plasma membrane. This ultimately culminates in “danger” signals being communicated to uninfected recipient cells, as discussed below.

Another viral protein examined for possible contribution to EV biogenesis was ICP0. We primarily focused on ICP0 due to its roles in autophagy evasion and endocytosis. ICP0 is known as a “promiscuous transactivator,” enhancing the expression of genes introduced into the cells by infection or transfection (1, 101–104). It is also an E3 ubiquitin ligase that targets host proteins for proteasomal degradation (105–107). Encoded by an immediate early gene, ICP0 localizes to the nucleus after production, where it degrades components of the nuclear domain 10 (ND10) bodies, dispersing cellular repressors of viral DNA to activate viral gene transcription (1, 101–103, 105–119). The onset of viral replication triggers ICP0 translocation to the cytoplasm, where its roles are less understood (120). We recently discovered that during HSV-1 infection, ICP0 is involved in degrading several autophagy adaptor proteins, including the p62/SQSTM1 (sequestosome 1) and OPTN (optineurin), independently of its E3 ubiquitin ligase activity (121). Cytoplasmic localization of ICP0 is essential for eliminating these adaptor proteins, observed as soon as 3 h post-infection (121). Autophagy is not involved in the elimination of these adaptors, as the WT virus effectively blocks autophagy in these cells (97, 99). Additionally, at a later time point, a fraction of these adaptors is exocytosed (40). We believe that this ICP0 function, along with the exocytosis of LAMP1 and lipidated LC3, serves as a complementary mechanism of the virus to evade autophagy.

Further insights into the cytoplasmic role of ICP0 were obtained through a yeast two-hybrid screen, which revealed that ICP0 interacts with the Cbl-interacting protein of 85 kDa (CIN85) (122). CIN85 belongs to a family of adaptor proteins with established roles in the spatial and temporal assembly of protein complexes during receptor endocytosis (123–128). It contains three N-terminal SH3 domains, a central proline-rich region, and a C-terminal coiled-coil domain (123–128). Through its SH3 domains, CIN85 recognizes a consensus Px(*P*/A)xxR motif present in several proteins involved in endocytosis (123–128). These interactions suggest that CIN85 is a central adaptor, playing a role in assembling endocytic machineries for surface receptor internalization and suppressing surface receptor signaling (123–128). ICP0 has four putative CIN85 SH3 binding motifs, three of which are located between amino acids 245–395 (122). A GST-fusion form of ICP0 (245–395 aa) can pull-down CIN85 from cell lysates (122). This interaction promotes downregulation of both the total and surface levels of the epidermal growth factor receptor (EGFR) in the absence of EGF ligand (122). Thus, it was proposed that ICP0, along with CIN85 and Cbl, negatively regulate receptor tyrosine kinases (RTKs) (122). Since RTKs are involved in host defense, inflammation, cell survival, and autoimmunity, the virus-induced reduction of their surface abundance could serve as an immunoevasion mechanism. Additionally, the ICP0/CIN85/Cbl complex promotes the reduction of the virus entry receptor Nectin-1 from the surface of infected cells, increasing the likelihood of entering uninfected cells (129).

These two cytoplasmic functions of ICP0 prompted us to investigate its potential role in EV biogenesis. We found that an ICP0-null virus cannot stimulate EV biogenesis compared with the WT virus; thus, the number of EVs from ΔICP0 infected cells is comparable with that of uninfected cells (40). Furthermore, markers found in EVs from HSV-1-infected cells were present in lower amounts in EVs from ΔICP0-infected cells (40). Although the ΔICP0 virus partially loses the ability to evade autophagy, the EVs from ΔICP0-infected cells do not resemble those from ΔICP34.5-infected cells (40). Expression of ICP34.5 by the ΔICP0 virus likely prevents microvesicle production. We conclude that ICP0 is required for optimal EV biogenesis in productively infected cells. Supporting this, ongoing studies in our lab using a mutant virus where ICP0 cannot interact or colocalize with CIN85 showed that endosomal cargo associated with the CIN85 structures cannot be exocytosed. This mutant virus also failed to evade type antiviral responses, partly due to an inability to promote exocytosis of autophagy and innate immunity components.

In addition to ICP34.5 and ICP0, several other viral proteins interact with membrane trafficking pathways, although their exact roles in EV biogenesis remain unclear. For example, vacuolar protein sorting-associated protein 4 (Vps4), a component of ESCRT machinery, is essential for HSV-1 cytoplasmic envelopment (85, 87). Vps4 is a type I AAA-ATPase that leads to the disassembly of ESCRT-III following membrane fission (130). Cells expressing the dominant negative form of Vps4, Vps4EQ, displayed only a modest defect in capsid accumulation to the cytoplasm, but these capsids remained unenveloped (85, 87). Although Vps4 is likely required to produce CD63+ and ESCRT+ EVs during infection, this needs confirmation.

The ESCRT machinery is also recruited by the nuclear egress complex (NEC), including UL31 and UL34 proteins, to the inner nuclear membrane for the primary envelopment of the virus. Loss of ESCRT-III proteins, such as CHMP4 or Alix, results in virion entrapment in the perinuclear space, likely due to scission failure (25, 82–84, 88, 131).

Another viral protein of particular interest is UL36, an essential inner tegument protein of the HSV virion (87). Lack of UL36 results in the accumulation of mislocalized, non-enveloped capsids docked to organelles, possibly due to the absence of tethering/targeting functions (87). Given UL36’s conserved role in capsid envelopment across Herpesviridae, it is considered a good candidate in the recruitment of ESCRT (83, 132). Additionally, its deubiquitinase activity may help remove ubiquitin from proteins sorted through ESCRT (83, 132).

Other tegument and envelope proteins, including the tegument proteins U_L_7, U_L_11, U_L_16, U_L_21, U_L_46, U_L_47, U_L_48, U_L_51, Us9, and the heterodimers of the envelope proteins gM/gN, gK/UL20, and gE/gI, likely interact with ESCRT and other membrane trafficking molecules (25, 82–84, 88, 131). The contribution of these viral proteins to the biogenesis of particles released by infected cells is also under investigation. L-particles and the ESCRT+ EVs, which are structurally simpler and contain fewer virion components, may help identify sites of capsid docking to organelles and of virion assembly, as well as delineate the scission events. Conversely, the increased production of CD63+ EVs suggests that the infection activates the biogenesis of these particles, although CD63 does not play an apparent role in HSV-1 envelopment, as capsids do not colocalize with CD63 (32). This adds a layer of complexity and indicates that viral products can modulate membrane reorganization and trafficking pathways even if they are not directly involved in virion assembly.

## SEPARATION OF DIFFERENT TYPES OF PARTICLES FROM HSV-1-INFECTED CELLS

**Fig 3:**
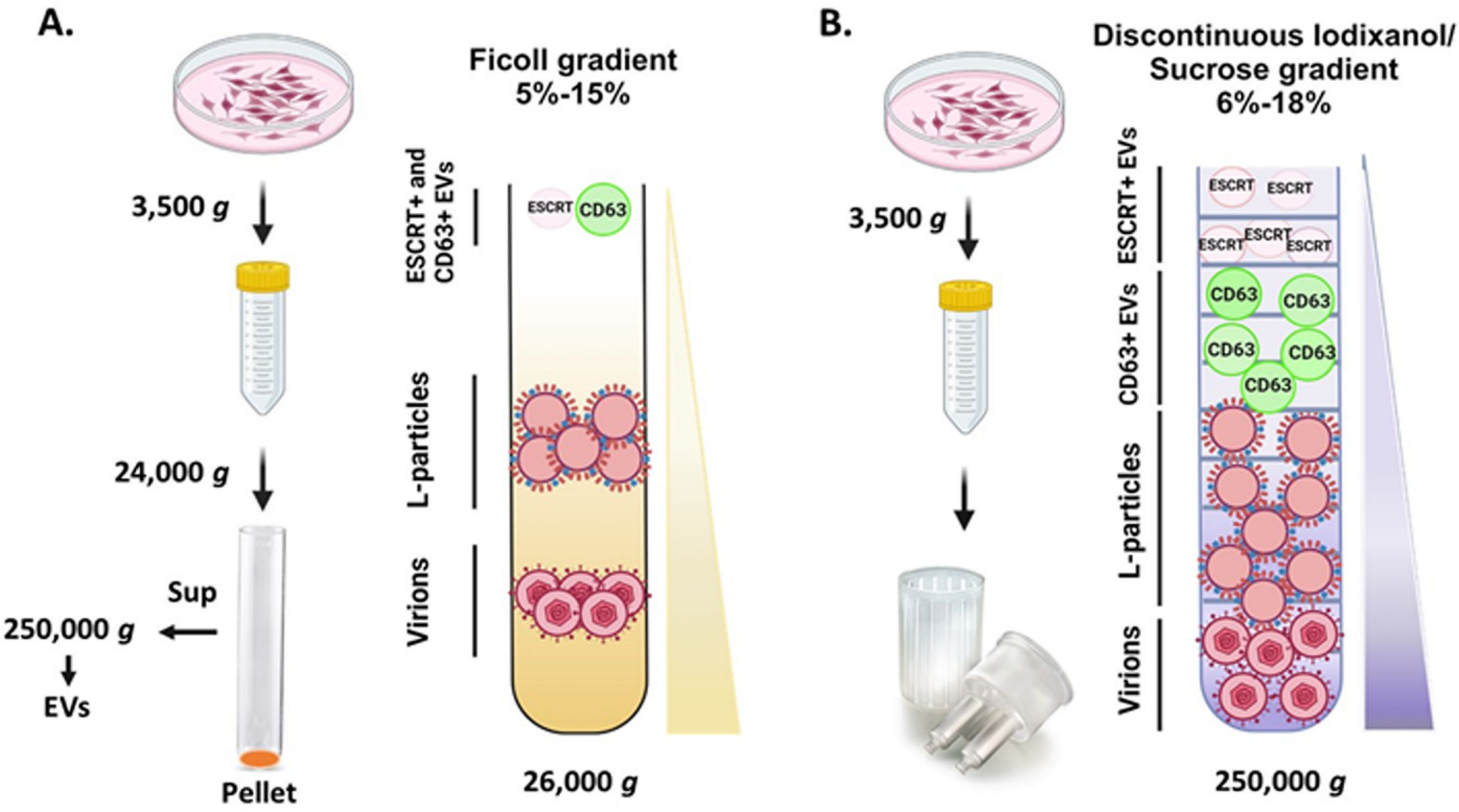
Procedures to separate different populations of particles released by HSV-1-infected cells. (A) Separation of L- from H-particles was achieved through a continuous 5%–15% Ficoll gradient (38). The supernatant of infected immortalized fibroblasts (hTERT-HEL) or the spontaneously immortalized retinal pigment cells (ARPE-19) was clarified through a low-speed centrifugation (3,500 × *g*) to remove floating cells and cell debris followed by an ultracentrifugation (24,000 × *g*) to pellet virions and other particles. This pellet was loaded onto the top of a continuous 5%–15% Ficoll gradient, span at 26,000 × *g,* and 500 µL fractions were collected from the top to the bottom of the gradient. The supernatant was spun at 250,000 × *g* and analyzed for different particles as well. (B) Separation of ESCRT+ EVs, CD63+ EVs from L and H particles was achieved through a discontinuous 6%–18% iodixanol/sucrose gradient (1.2% increments) (39, 64, 65). The supernatant of cultures infected as above was clarified by low-speed centrifugation, followed by filtration through 0.45 µm filters and concentration through 100 kDa cutoff filters. The filtered and concentrated supernatant was loaded onto the top of a discontinuous 6%–18% iodixanol/sucrose gradient followed by centrifugation at 250,000 × *g*. Fractions (500 µL) were collected from the top to the bottom of the gradient and analyzed for markers of different particle populations including infectious virions. Ultracentrifugation was performed using a SW41 rotor.

Efficient procedures have been developed to separate infectious (Heavy-H) from non-infectious (Light-L) HSV-related particles, based on density differences between the L-particles (lacking capsid and viral genome) and H-particles (38). L-particles average 140 nm in size, whereas H-particles are about 180 nm by comparison. During sedimentation of pelleted extracellular particles (24,000 *g*) from infected culture supernatants through a 5%–15% continuous Ficoll gradient, two main bands are observed: a diffuse upper band and a sharp, well-defined lower band (Fig. 3A) (38). This separation reflects significant density differences between the two particle types. The sharp lower band indicates homogeneity of H-particles, whereas the diffuse upper band reflects the heterogeneity of L-particles (38). Notably, ESCRT+ and CD63+ EVs markers are absent from the L- and H- particle bands but are enriched in the supernatant after centrifugation (24,000 × *g*), further supporting the distinction between EVs and L-particles (Fig. 3A).

We developed a procedure to separate the EVs from the virions and the L-particles by leveraging their density differences using a discontinuous iodixanol/sucrose gradient (iodixanol concentration ranging from 6% and 18%, in 1.2% increments) (Fig. 3B). Concentrated supernatant from infected cultures (100 kDa cutoff filter devices) was processed with low-speed sedimentation (up to 3,500 × *g*) to avoid particle co-aggregation. ESCRT+ EV markers, along with a few tegument and envelope proteins of the virus, were found within the first four gradient fractions (500 µL fractions), whereas CD63+ EVs were found in deeper fractions (spread between fractions 5 or 6, and all the way to fraction 12) without viral proteins (39). Both types of particles have heterogeneous sizes (50–400 nm, peak at 120 nm) (39). Capsid proteins such as UL38 (used as markers of intact virions) were found in the bottom four fractions of the gradient, whereas tegument and viral envelope proteins spread between fractions 11 and 12 and toward the bottom of the gradient, indicative of heterogeneous capsid-less particles enriched in virion components, likely L-particles (39).

Although gradient-based methods help identify distinct particle populations, the presence of overlapping markers across fractions necessitates further testing to confirm the origins of individual particles (to distinguish if they belong to the same or different particle populations). Investigations are underway to determine whether viral components are present in the ESCRT+ EVs.

On the other side, it is of equal importance to consider host protein incorporation in virions. Several host proteins have been identified in purified HSV-1 virions. These include but are not limited to (i) the annexin family of proteins that regulate membrane dynamics and signaling, (ii) ADP-ribosylation factors (ARF) that function as GTP regulatory proteins, (iii) Ras-associated binding proteins (Rab) that are involved in membrane bending, vesicles targeting and fusion, and (iv) the kinesin-1 protein that takes part in motorizing virions (133, 134). Although these host proteins could be incorporated into the tegument and envelope of virions, highly purified virions are essential to validate these findings, as sedimentation techniques may cause particle co-aggregation, leading to potential data misinterpretation if not rigorously sorted out. Complementary approaches are also recommended to verify results.

Conventional EV isolation methods (ultracentrifugation, precipitation, and size exclusion chromatography) ensure high purity and minimal damage, but resolution is low due to the heterogeneity of EV populations (135–139). Advanced methodologies have since been developed to resolve these issues, in order to more reliably separate distinct EV populations (135–139). Immunoaffinity approaches, for example, offer a very robust way to isolate populations at higher resolution if surface EV markers are known. Microfluidic approaches, on the other hand, account for the physical nature of EVs, including their size, volume, viscoelasticity, and electrochemical properties. Although these advanced methods alone offer additional benefits, greater resolution and purity can further be achieved by employing a combination of approaches. For instance, combining tangential-flow filtration, polyethylene glycol (PEG) precipitation, and multimodal chromatography (MMC) can separate particles by size and binding properties, as found in a recent study (140). These combined approaches highlight the potential for refining EV isolation.

Overall, our research highlights that infected cells release several types of particles, each with distinct molecular, biochemical, and biophysical characteristics. Although some of these particles can be separated using gradient-based approaches, others require customized methods tailored to specific markers.

## CARGO AND FUNCTIONS OF PARTICLES RELEASED DURING HSV-1 INFECTION

The discovery of the L-particles has generated questions about their potential role during HSV-1 infection. L-particles enter the cells through the same receptor and utilize similar mechanisms for attachment, fusion, and tegument protein release as the H-particles (33). This similarity lends to question if L-particles antagonize virus entry. However, to the contrary, they have been found to increase HSV-1 DNA infectivity, by supplying tegument proteins required for initiating viral gene transcription, such as VP16 and its cofactors UL46 and UL47 (35). Additionally, L-particles can partially complement defective co-infecting virions. For instance, the virion host shutoff (vhs) protein when incorporated into L-particles is fully functional and enhances infectivity (141). Since vhs is used by the virus to evade immune responses by degrading host mRNAs, L-particles entering uninfected cells could prime these cells to enhance a subsequent infection (1, 142). Furthermore, L-particles entering latently infected cells could provide the tegument proteins required for latency reactivation, although this has not been explored thus far. Another proposed function of L-particles is that they act as decoys for the immune system by binding specific antiviral antibodies, thereby reducing the effectiveness of adaptive immune responses (33). Other mechanisms of immunoevasion by L-particles have been proposed. For example, H-particles of different alphaherpesviruses have been shown to stimulate high levels of IFN-α production in plasmacytoid dendritic cells (143). The H-particle-induced IFN-α production was suppressed by L-particles of both PRV and HSV-1, in a gD-dependent manner (143). Antibodies against gD, but not against gB or gC, interfere with HSV-1-induced IFN-α production by peripheral blood mononuclear cells (143). It is possible that L-particles antagonize H-particles for binding to a receptor(s) required for IFN-α production. Additional studies have demonstrated that HSV-1 L-particles released from human monocyte-derived mature dendritic cells (mDCs) induce CD83 downregulation in uninfected bystander mDCs (144). CD83 is critical for regulating and resolving immune responses and plays an essential role in differentiating T and B lymphocytes. Similarly, HSV-1 infection of mDCs downmodulates the expression of the IL-6 receptor (IL-6R), and L-particles released from HSV-1-infected mDCs further reduces IL-6R expression in uninfected bystander mDCs, thereby suppressing IL-6 signaling (145, 146). Also, there is evidence suggesting that L-particles may contribute to amyloid plaque formation (147). The 42-amino-acid form of amyloid-β (Aβ42) plays a key role in the pathogenesis of AD and serves as the primary biomarker for AD diagnosis (148). Several tegument proteins present in L-particles, along with the envelope protein gC, appear to interact with Aβ42, promoting the formation of insoluble amyloid plaques, a key characteristic of AD (147). Also, the VP22 protein in L-particles may contribute to AD pathogenesis due to its antigenic similarity to the Tau protein, which promotes the formation of neurofibrillary tangles during AD (147).

The effects of ESCRT+ EVs on HSV-1 infection resemble those of L-particles (39). When cells are exposed to ESCRT+ EVs prior to a low PFU infection, we observed an increase in viral replication (39). This effect was attributed to viral proteins being delivered by the ESCRT+ EVs, such as the VP16 transactivator (39, 65). Notably, VP22, UL46, gM, and several other tegument and envelope proteins that were present in L-particles were absent from the ESCRT+ EVs, thus their ability to evade antiviral responses remains to be elucidated. The glycoprotein gB, which co-fractionates with ESCRT+ EVs, has been shown to disrupt MHC-II surface expression in EV recipient cells, potentially interfering with the activation of adaptive immune responses against HSV-1 (149). Intracellularly, gB is sorted to MVBs, where HSV-1 envelopment and egress occur, and a fraction of gB may be exocytosed via this pathway (61). In addition to gB, the ESCRT+ EVs carry all other components of the virus entry machinery, including gD, gH, and gL. However, like L-particles, these EVs do not appear to antagonize viral entry into the cells (39). This is in contrast to EVs released by EBV-infected cells, which carry the EBV glycoprotein gp350. The gp350 binds to the CD21 (cluster of differentiation 21) receptor on the B cells and inhibits the spread of EBV to bystander uninfected B cells, at least *in vitro* (150). Additionally, using a targeted approach, we did not detect the activation of type I IFN and pro-inflammatory responses by the ESCRT+ EVs. Since the ESCRT+ EVs carry gD, it is important to determine whether they can block H-particle-induced IFN-α production in plasmacytoid dendritic cells, similar to L-particles. It is also important to determine if the ESCRT+ EVs downmodulate IL-6R expression in the uninfected recipient cells, like L-particles do. Supporting these findings, an *in vivo* study determined that EVs present in the tears of individuals with recurrent herpes simplex keratitis (HSK) contain viral products that likely facilitate viral spread during HSK recurrence (151).

Unlike the ESCRT+ EVs and the L-particles, the CD63+ EVs have a negative effect on HSV-1 infection (39). These EVs lack viral factors but contain host factors with antiviral activity, such as the STimulator of INterferon Genes (STING) (39, 152, 153). STING is a DNA sensor that localizes on the endoplasmic reticulum (ER). Upon binding non-canonical cyclic dinucleotides, such as 2΄ 3΄-cGAMP, STING translocates to the TGN, where it oligomerizes and acts as an adaptor to activate TANK-binding kinase 1 (TBK1) and downstream type I IFN signaling pathways (154–158). However, treatment with 2΄ 3΄-cGAMP or infection with a replication-deficient mutant is insufficient to induce STING exocytosis, although STING dimers are formed, indicating that innate immune activation is not enough for STING exocytosis. Consistently, STING exocytosis was observed in HSV-1-infected IFNAR1 KD cells. It was discovered that STING exocytosis requires viral late gene expression and occurs in a CD63-dependent manner (39, 152). STING is exocytosed by infected cells primarily in dimeric and other oligomeric forms (39, 152). Additionally, STING trafficking through the Golgi and its palmitoylation are essential for its exocytosis. It is thought that palmitoylated STING may co-clusters with palmitoylated CD63, possibly through lateral association in the TGN and/or MVBs, and this association may facilitate STING exocytosis. EVs from STING-expressing cells trigger antiviral responses, inhibiting a subsequent HSV-1 infection. In contrast, EVs from STING KD cells do not interfere with the progression of a subsequent infection (39, 152). Furthermore, the inhibition of IFNAR1 in EV recipient cells does not affect the ability STING-containing EVs to restrict a subsequent HSV-1 infection. Although exosomal STING can directly activate antiviral responses in recipient cells, STING-dependent factors delivered with the EVs could also activate these responses. Several cytokines and chemokines could be packaged within EVs and contribute to the priming of recipient cells. Remarkably, however, donor-infected cells producing STING-containing EVs do not exhibit antiviral responses themselves, as the virus effectively counteracts these responses, preventing the production of cytokines or chemokines (39, 152). This indicates that STING exocytosis in productively infected cells likely represents a viral immunoevasion strategy to eliminate STING from infected cells. However, recipient cells are likely primed and exert a negative effect on the infection. These phenomena may be due to the coevolution of the virus with the host, where the host is protected by facilitating the establishment of a viral latent reservoir. STING exocytosis has also been observed in other herpesviruses, including VZV and HCMV, which similarly induce CD63 exocytosis (152). For many viruses, 2΄ 3΄-cGAMP is packaged both in virions and EVs and can activate antiviral responses in recipient cells (159, 160). This likely occurs in cells where the viruses cannot effectively counteract antiviral responses. However, in human fibroblasts and epithelial cells, where HSV-1 effectively blocks antiviral responses, including cGAS activity, significant amounts of 2΄ 3΄-cGAMP are unlikely to be present in HSV-1 virions or EVs. Supporting this, EVs from HSV-1-infected STING KD cells cannot activate antiviral responses in recipient cells. Finally, Sp100A, a component of the nuclear domain 10 (ND10) bodies, has been found in EVs released by HSV-1-infected cells and exhibited a negative effect on the infection (161). Sp100A is induced by IFNs and accumulates on both host and viral promoters, regulating gene expression. However, the specific population of EVs that carry Sp100A has yet to be determined.

Notably, we also found that total EVs derived from infected fibroblasts activated an antiviral response when exposed to the human leukemia monocytic cell line, THP1. These EVs stimulated the expression of IFN-β1, ISGs (ISG56, ISG15), and pro-inflammatory cytokines IL-6 and IL-1β, but not IL-12 or TNF-α (64). Additionally, the EVs upregulated CCL2, a chemotactic cytokine that recruits immune cells (64). A shift toward an M1-like macrophage phenotype was also observed, which likely activates pathways to suppress HSV-1 infection and limit viral spread while minimizing host damage from inflammation (64). Several of these cytokines and chemokines have been shown to control the viral spread in different model systems and to recruit immune cells, including NK and T cells (162).

Although ESCRT+ EVs exert a positive effect on the infection and CD63+ EVs exert a negative effect, we found that the net effect of EVs present within the first 10 fractions of our iodixanol/sucrose gradient was overall negative for the infection (39). This net negative effect may result from the higher abundance of CD63+ EVs compared with ESCRT+ EVs, and it is possible that this net effect reflects these numerical differences (39). Alternatively, the cargo composition of the EVs could play a part in the overall net effect. Since viral infection produces an array of different particle types, their combined effects will depend on a variety of factors such as their overall abundance, the timing of their production during infection, their cargo, their cell targets, as well as the host responses.

In contrast to EVs from WT virus-infected cells, EVs produced after infection with mutant viruses either did not carry STING (EVs from ΔICP0 infections) or they carried a short, modified form of STING (EVs from ΔICP34.5 infections) (40). These mutant viruses were unable to counteract antiviral responses, and their EVs mounted stronger antiviral responses in recipient cells compared with the EVs from WT virus-infected cells (40). The composition of cytokines, chemokines, and other antiviral factors in these mutant-virus EVs remains to be determined.

Multiple studies have provided evidence that small-sized virions, such as those from most RNA viruses, can be packaged in EVs (50–52, 54, 60, 163, 164). Regarding herpesviruses, human herpesvirus-6 (HHV-6) appears to induce the formation of MVB-like vacuoles and egress through the MVBs by an exosomal release pathway (165). This raised the question of whether HSV-1 virions could also be packaged in EVs. Through the gradient approaches described earlier, we found that viral DNA is absent from the fractions containing ESCRT components or the tetraspanin CD63, indicating that HSV-1 virions are not packaged in these EVs. However, studies using a human oligodendroglia cell line revealed that upon HSV-1 infection, virions could be detected in microvesicles produced by these cells (166). Furthermore, it was shown that the Chinese hamster ovary (CHO) cell line, which is resistant to infection by free HSV-1 virions, became susceptible to HSV-1 infection following exposure to virus-containing microvesicles (166). These findings suggest that packaging of HSV-1 virions into EVs might occur in a cell type-dependent manner. It is also possible that a small fraction of microvesicle-encapsulated virions are always present in infected cultures, and they co-fractionate with L-particles (or virions in the density gradients), and for this reason, they may have been overlooked.

In addition to the proteinaceous cargo, EVs produced by HSV-1-infected cells also carried viral transcripts (153). Among the transcripts found in EVs were VP16, the latency-associated transcript (LAT), and several viral miRNAs (153). Although LAT and the viral miRNAs may prime the uninfected cells for latency establishment, VP16 expression drives lytic gene expression. Thus, the outcome of a subsequent infection likely depends on the relative amounts of these transcripts delivered to recipient cells. The specific EV population carrying the viral transcripts, the quantities of viral transcripts into EVs, and the mechanism of their packaging into these EVs are under investigation. Supporting these findings, selected viral miRNAs were recently detected in EVs derived from the cerebrospinal fluid of HSV-1 patients with neuroinflammation (167). Larger cohort studies are required to better characterize the viral cargo present in EVs under disease conditions.

The most challenging aspect of EV biology is to determine the functions of EVs. This is because (i) EVs are highly heterogeneous; thus, it is difficult to define the different EV populations, particularly since specific markers to define these populations are lacking; (ii) EV biogenesis is a dynamic and complex process, where disruption of one EV biogenesis pathway results in the biogenesis of different EV populations via newly activated, likely compensatory and/or redundant pathways; (iii) the target cells of the different EV populations remain unknown; (iv) determining a biologically relevant dose of EVs is difficult, as cells constantly release and uptake EVs; (v) EV cargo is highly variable and depends on multiple factors such as the timing of EV collection, the donor cell status, and culture conditions; (vi) systems to track the trafficking and uptake of EVs *in vivo* are primitive; (vii) EV isolation, purification, and preservations methods can impact EV content and stability; and (viii) viruses hijack membrane trafficking pathways for envelopment and egress, and this impacts EV biogenesis pathways, resulting in the production of non-canonical and unconventional EVs. Despite these challenges, understanding EV biology is crucial for uncovering their roles in health and disease. A multipronged approach should be used to mitigate some of these challenges during a viral infection that incorporates: (i) a comprehensive EV cargo approach using multi-omics across different model systems to identify conserved EV cargo, infection/disease-specific cargo, and cell type-dependent cargo; (ii) utilizes higher resolution isolation approaches, including SEC, microfluidic devices, and immunoaffinity to identify and isolate the most abundant particle populations during an infection; (iii) utilizes simple and relevant *in vitro* and *in vivo* models to determine signaling responses of different EV populations and their impacts on the infection and the host; (iv) quantifies EVs and relevant cargo to determine a biologically relevant dose; (v) identifies host EV biogenesis factors subjugated by the virus for envelopment and egress, as they could have a therapeutic potential; (vi) and identifies viral genes that subjugate EV biogenesis pathways.

## EXTRACELLULAR VESICLES DURING HSV-1 INFECTION AND CLINICAL IMPLICATIONS IN NEUROPATHOGENESIS, ONCOLYTIC VIROTHERAPY, AND VACCINE DEVELOPMENT

### HSV-1-derived EVs and their role in neuropathogenesis

HSV-1 maintains its latent reservoir within sensory neurons of the peripheral ganglia and in the CNS, particularly in elderly individuals. “Clinically silent” reactivation and replication of the virus in the CNS occurs periodically and has been linked to sporadic Alzheimer’s disease and other neurodegenerative disorders, including Parkinson’s disease and schizophrenia. Therefore, it is critically important to determine how different types of particles released by HSV-1-infected cells impact neuronal functions. Recent studies demonstrated that following HSV-1 infection, there is increased phosphorylation of tau (tubulin-associated unit), a protein involved in microtubule stability in axons. Hyperphosphorylation of tau leads to its aggregation in neurofibrillary tangles, which disrupt neuronal functions. It was also discovered that high molecular weight isoforms of phosphorylated tau (p-tau T181, p-tau T205, and p-tau T217) are released via EVs (168). The EVs carrying p-tau are then taken up by other cells inside the brain, where they promote misfolding and aggregation of native tau (168). In support of these findings, it was reported that EVs isolated from CSF of AD patients contain tau seeds that favor tau aggregation in cultured cells and trigger expression of neurodegeneration-associated genes (169, 170).

Additionally, HSV-1 glycoprotein G (gG) has been shown to modify the cargo of EVs produced by infected cells, promoting neurite outgrowth (171). This effect was observed even when gG is expressed outside the context of the infection. It was then proposed that gG is responsible for enriching EVs with galectin-1 (171). Galectin-1, in turn, promotes neurite outgrowth by stimulating the expression of genes associated with neurite extension and contributes to axonal regeneration following nerve injury.

A recent study analyzed exosomal miRNAs in the CSF of HSV-1 patients to determine whether active, reactivated, or latent HSV-1 would play a role in subarachnoid hemorrhage (SAH), psychiatric disorders (affective and schizophrenic spectrum), and other neuroinflammatory conditions (167). The study found elevated levels of miR-H27 in nearly all samples, indicating the presence of replicating HSV-1. Tissue damage and inflammation in SAH patients and other neuroinflammatory conditions likely trigger HSV-1 reactivation. In patients with schizophrenic disorders, miR-H3-3p was particularly elevated, indicating that a latent HSV-1 reservoir is present and likely contributes to this condition (167). Additionally, miR-155–5p levels correlate with the level of neuroinflammation, whereas miR-138–5p suggested that EVs may originate from brain-infiltrating leukocytes (167). These findings demonstrate that miRNA profile in CSF exosomes can provide valuable insight into HSV-1 infection status and neuroinflammatory activity.

Finally, we and others have discovered that EVs released by HSV-1-infected cells carry the essential viral transactivator VP16, along with the host transcription factor Oct-1 (octamer transcription factor-1). Oct-1 recruits VP16 to the immediate-early promoters of the virus to enable their expression (39, 172, 173). This cargo likely promotes virus replication. Other viral proteins present in EVs, including Us11 and vhs, could evade RNA sensing pathways, further promoting the infection. In contrast, EVs carrying antiviral factors such as STING, along with viral miRNAs expressed during latency, have been shown to suppress viral activity. As previously discussed, the ratio of the proviral versus antiviral factors in EVs determines the outcome of the infection.

Overall, it is important to understand how HSV-1-associated EVs disrupt neuronal functions and activate the neurodegeneration cascade. These findings will help unveil novel therapeutic targets to mitigate virus-driven neuropathogenesis.

### HSV-1-based oncolytic virotherapy and potential roles of EVs

Two oncolytic virotherapies based on herpes simplex virus one strains (oHSV) have been approved for clinical use: Talimogene laherparepvec (T-VEC) for the treatment of unresectable metastatic melanoma and the G47Δ, conditionally approved in Japan for glioblastoma following a successful phase II clinical trial (174, 175). These oncolytic viruses lack both copies of the *ICP34.5* gene, preventing replication in normal tissues, but allowing replication in certain tumors. Both oHSVs have additional modifications, including deletions and/or gene insertions, to optimize their oncolytic potential and increase their safety profile (174, 175). The recommended dose for both oHSV therapies is at least 10^8^ PFU per injection, often requiring multiple injections for effectiveness. However, stocks of these viruses are expected to contain at least 100–1,000× more EVs per PFU, based on quantification of EVs released by ΔICP34.5 virus-infected cells, along with a substantial number of L-particles. These EVs could activate robust innate immune responses and pro-inflammatory pathways in recipient cells, potentially enhancing the oncolytic potential of these viruses by recruitment and activation of immune cells. However, it was discovered in multiple studies that EVs from HSV-1-infected cells also carry viral glycoproteins gB and gH, which interfere with MHC II surface localization in recipient cells, a means of effectively inhibiting T-cell responses (149). This effect could negatively impact both the safety profile and the oncolytic properties of these viruses due to adaptive immune suppression. Conversely, to sustain the function of MHC I, the *ICP47* gene was deleted from both viruses to increase their oncolytic potential (174, 175). ICP47 prevents TAP (transporter associated with antigen presentation) from transporting peptides into the ER lumen, where they are loaded onto MHC I molecules (176). In the absence of ICP47, CD8+ T cell responses can be activated (176). Perhaps, then, future generations of oncolytic viruses could be designed to mitigate the impact of gB on MHC II trafficking, preserving adaptive immune responses against tumor cells and further improving therapeutic outcomes.

A similar scenario has been proposed for other viruses that are currently being evaluated for oncolytic therapy of human melanoma. These include the vesicular stomatitis oncolytic virus (VSV-OV), the vaccinia oncolytic virus (VACV-OV), and reoviruses. VSV OVs and VACV OVs were found to increase EV production when tested in different melanoma cell lines (177). These EVs (OV-EVs) were enriched in immunity-related proteins, such as the human leukocyte antigen (HLA) class I molecules (HLA-ABC), β2-microglobulin, and the melanoma antigen Melan-A. These EVs enhance the cytotoxic activity of human CD8+ T cells, augmenting their anti-tumor properties (177). By analogy, a phase III clinical trial in melanoma patients demonstrated that local injections of the G47Δ oHSV not only targeted injected tumors but also activated systemic antitumor immunity. It is therefore possible that EVs released by G47Δ-infected tumor cells play a contributing role in activating this systemic antitumor immunity.

Reoviruses have also been investigated as potential oncolytic agents, due to their high cytotoxicity in certain tumors and spontaneously transformed cell lines. These viruses are released from infected cells either in association with or encapsulated within small extracellular vesicles (SEVs) and/or a lipid bilayer-like structure (178). In addition to virions, SEVs contained various immunostimulatory components, including damage-associated molecular patterns (DAMPs), such as IFN-β, HSP70, HSP90, HMGB1, pathogen-associated molecular patterns (PAMPs) like the outer capsid protein σ3, and tumor antigens (178). SEVs released from reovirus-infected cells (B16 melanoma) enhance tumor cell killing, innate immune responses, and CD8^+^ T cell tumor infiltration. These effects result in greater tumor size reduction compared with reovirus infection alone (178).

### HSV-1 EVs and potential role in vaccine development

EVs possess several features that make them promising candidates for vaccine development. They have a low basal immunogenic profile; they preserve the naïve conformation of antigens and can circulate through body fluids to reach all organs. Additionally, EVs may accumulate at sites of infection due to the enhanced permeability and retention (EPR) effect, and therefore, it is feasible to scale up their production (179–181). The potential of EVs is further amplified by the ability to engineer them to package specific antigenic cargo.

EVs released by infected cells naturally carry viral antigens and have been considered potential therapeutic agents for EV-based vaccine development. For instance, it has been shown that CD63+/CD81+ EVs released from monocytes infected with influenza virus, EBV, or HCMV are loaded with viral peptides and can trigger the release of IFN-γ from CD8+ T cells in an antigen-specific manner (182). Several mechanisms of antigen presentation involving EVs have been described. For example, EVs are taken up by DCs which present the entire antigenic peptide-MHC complex from EVs to T cells. Alternatively, DCs can uptake EVs and they present EV peptides on their own MHC I and MHC II molecules. Finally, EVs carry MHC I and MHC II on their surface, and they can directly activate CD4+ or CD8+ T cells by presenting peptides (183).

The EVs released by HSV-1-infected cells could be used for vaccine development as they carry several viral antigens, including the envelope glycoproteins gD, gB, gH, and gL. Furthermore, gB from several alphaherpesviruses disrupts the trafficking of MHC II to the plasma membrane and instead increases its sorting to extracellular vesicles (149). This highlights the critical need to identify the peptides presented by MHC II on EVs during HSV-1 infection to determine whether these EVs function as immune decoys or activate adaptive immune responses against the virus. Finally, it is important to determine whether HSV-1 gE is present in EVs released by infected cells and whether it exerts its FcγR function. HSV-1 gE functions as an IgG Fc receptor (FcγR) blocking the Fc domain of human antibodies targeting the virus or infected cells. HSV-1 FcγR blocks both IgG Fc-mediated complement activation and antibody-dependent cellular cytotoxicity, which could impact the immune response and vaccine efficacy (184, 185).

## IS THE PRODUCTION OF EVS A VIRUS- OR HOST-INDUCED PROCESS?

The mechanisms of EV production during HSV-1 infection can provide clues of whether this is a virus- or host-driven process. Our findings regarding the requirements for EV biogenesis by infected cells can be summarized as follows:

1. EV production is stimulated by virus late gene expression, replication, and likely primary envelopment. Although a replication-deficient virus can also stimulate EV biogenesis, these EVs differ by lacking the CD63 tetraspanin and are likely generated through different biogenesis pathways (39, 65).
2. Exposure of cells to non-canonical cyclic dinucleotides, infection with UV (ultraviolet light)-inactivated virus, or mutants incapable of evading antiviral responses do not promote STING exocytosis (152). Additionally, neutralizing the IFNR1 receptor does not interfere with STING oligomerization or exocytosis during HSV-1 infection (152).
3. Deletion of the *ICP34.5* gene compromises EV biogenesis through the CD63 exocytosis pathway but promotes the biogenesis of microvesicles from the plasma membrane (40). Viruses lacking *ICP0* produce similar levels of EVs as uninfected cells (40). Importantly, ICP0 mutants that fail to bind CIN85 result in the loss of selected cargo exocytosis.
4. Although the CD63+ EVs from WT virus-infected cells can activate antiviral responses in recipient cells, the donor cells fail to mount antiviral responses during a productive HSV-1 infection (39, 64, 152).
5. Some particles have a pro-viral effect in a subsequent HSV-1 infection (39).

Altogether, our findings support that the production of the CD63+ and ESCRT+ EVs from productively infected cells is induced by the virus. HSV-1 has evolved specific genes to evade host antiviral responses and control signals communicated by the infected cells. Comparison of EVs from productively versus non-productively infected cells will be useful in understanding how the virus diverts membrane trafficking pathways, promotes EV biogenesis, and controls cargo content.

## CONCLUDING REMARKS

For all known viruses, infection invariably results in the production of a variety of particles, ranging from intact/infectious virions and defective virus-like particles to chimeric EVs containing both host and viral factors, or EVs carrying host factors exclusively. For several viruses, *in vitro* findings have been validated *in vivo*, where similar particles have been detected in human-derived materials during the course of the viral disease. For HSV-1, L-particles were the first to be described alongside infectious virions. These L-particles lack a viral genome and capsid yet are produced in similar amounts comparable to infectious virions. HSV-1-infected cells also produce other particles via different EV biogenesis pathways. These particles, which carry viral and/or host factors are 10-fold to 100-fold more abundant than the infectious virions. The particles carrying viral factors were found to have a predominantly pro-viral role, whereas those lacking viral factors activated antiviral responses, negatively impacting the infection. Notably, there is evidence for repeated, low-grade HSV-1 infections in the brain of some infected individuals; therefore, it is possible that these particles contribute to chronic inflammation that could lead to neurodegeneration. The higher stability of EVs compared with free cargo could further prolong these effects. Overall, there is an urgent need to understand how these particles interact with the virus when they simultaneously enter the host, how they prime uninfected recipient cells, and how they impact virus transmission, the latent reservoir of the virus, and disease outcome.
